# The complete chloroplast genome of *Onosma fuyunensis* Y. He & Q.R. Liu and its phylogenetic analysis

**DOI:** 10.1080/23802359.2020.1861567

**Published:** 2021-10-08

**Authors:** Yi He, Xuemin Xu, Quanru Liu

**Affiliations:** Key Laboratory of Biodiversity Science and Ecological Engineering, Ministry of Education, College of Life Sciences, Beijing Normal University, Beijing, China

**Keywords:** Chloroplast genome, *Onosma fuyunensis*, phylogenetic analysis, Boraginaceae

## Abstract

The chloroplast genome sequences of Chinese Boraginaceae species, *Onosma fuyunensis* Y. He & Q.R. Liu, were reported in this study. We sequenced *O. fuyunensis* using the Illumina HiSeq X Ten platform. The total length of *O. fuyunensis* chloroplast genome is 150,612 bp, including a large single-copy region of length 82,853 bp, a small single-copy region of length 17,281 bp, and a pair of 25,239-bp inverted repeat regions. The chloroplast genome of *O. fuyunensis* has 133 genes, including 84 protein-coding, eight ribosomal RNA, and 37 transfer RNA genes. The overall GC content of the whole genome was 43.3%. The phylogenetic analysis revealed that *O. fuyunensis* is closely related to *Borago officinalis* and *Plagiobothrys nothofulvus.*

The genus *Onosma* L. belongs to the family Boraginaceae, it consists of ca. 240 species of biennial or perennial herbs and subshrubs. This genus is distributed in the temperate zones of the Old-World, with the main center of diversity in the Irano-Turanian region (Weigend et al. 2016). Many species of the genus *Onosma* have been reported as medicinal plants that contain rich bioactive compounds (Pandey 1970; Gu 2016; Sher et al. 2016). *O. fuyunensis* is a newly reported species distributed in Xinjiang, China (He et al. 2020). In this study, we sequenced the complete chloroplast genome of *O. fuyunensis*, provided the first chloroplast genome in this genus and tribe Lithospermeae.

The voucher specimens of *O. fuyunensis* were collected from Fuyun, Xinjiang Province, China (46°59′01″N, 89°41′42″E) and stored at Herbarium of Beijing Normal University (BNU, collected by Y. He & Y. Zhou, No. XJ133). The total DNA was isolated from approximately 0.05 g of dried leaves using the modified CTAB method. The extracted DNA was then purified using the Wizard DNA clean-up system (Promega Corp.). The complete chloroplast genome was sequenced at Novogene Biotech Co. (Beijing, China) using the Illumina HiSeq X Ten platform with a 150-bp shotgun library. Approximately 10 GB of 150-bp paired-end reads of *O. fuyunensis* were generated. The obtained raw data (NCBI accession number PRJNA673017), containing some low-quality short sequences, were processed and assembled as described by Bakker et al. (2016) and Wei et al. (2017). The sequencing results were mapped to the reference chloroplast genome sequence (*Salvia officinalis*, NCBI accession number NC_038165) using Geneious Prime 2020.2 (https://www.geneious.com) software. The complete chloroplast genome was annotated using DOGMA (http://dogma.ccbb.utexas.edu/) and Geneious Prime 2020.2 and checked manually. The circular chloroplast genome map was drawn in OGDRAW (Lohse et al. 2007). The complete chloroplast genome of *O. fuyunensis* (NCBI accession number MT341643) is 150,612 bp long and has a typical quadripartite structure, with one 82,853-bp large single-copy (LSC) region, one 17,281-bp small single-copy (SSC) region, and two 25,239-bp inverted repeats (IR) regions. It contained 133 genes including 84 protein-coding, 37 tRNA, and eight rRNA genes. In addition, six protein-coding, seven tRNA, and four rRNA genes are duplicated in the IR regions. Among annotated genes, eight protein-coding (*atp*F, *ndh*A, *ndh*B, *pet*D, *rpl*2, *rpl*16, *rpo*C1 and *rps*16) and six tRNA (*trn*A-UGC, *trn*G-UCC, *trn*I-GAU, *trn*K-UUU, *trn*L-UAA and *trnV-UAC*) genes contained one intron, while two genes (*clp*P and *ycf*3) contain two introns. The overall GC content of the chloroplast genome is 43.3%.

Eight complete chloroplast genome sequences were downloaded from the NCBI database to confirm the phylogenetic position of *O. fuyunensis*. Sequences were automatically aligned with MAFFT (Katoh 2002). Maximum-likelihood (ML) trees were performed with Randomized Axelerated Maximum Likelihood (RAxML)-HPC2 (Stamatakis 2014), from the Cyber-infrastructure for Phylogenetic Research (CIPRES) Science Gateway (Miller et al. 2010). The parameter settings followed Wei et al. (2017). *Forsythia suspensa* (NC_036367) was set as outgroup. The results showed that *O. fuyunensis* was closely related to *Borago officinalis* and *Plagiobothrys nothofulvus* (Figure 1).

**Figure 1.**
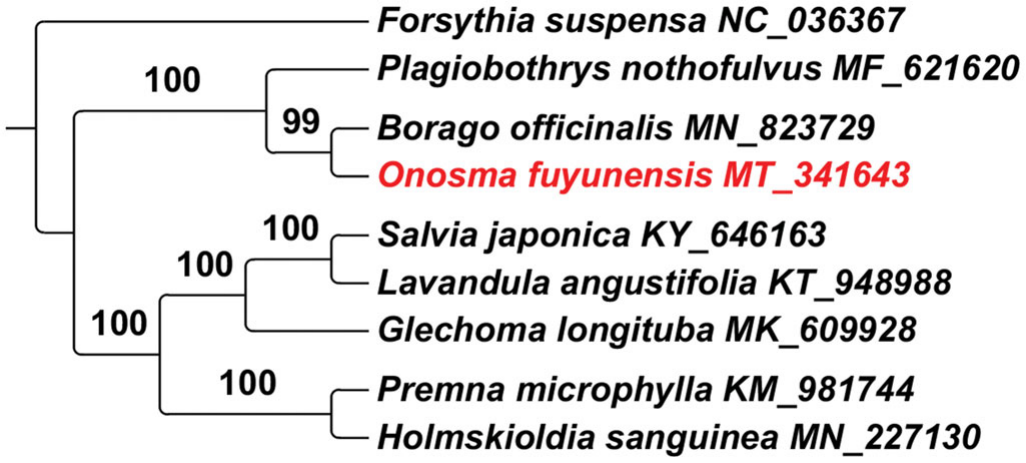
Phylogenetic tree of nine species based on the complete chloroplast genome by the maximum likelihood (ML) methods. Forsythia suspensa was the outgroup.

## Data Availability

The data that support the findings of this study are openly available in GenBank of NCBI at https://www.ncbi.nlm.nih.gov, reference number MT341643.
